# Supplementary material for: Combinatory Library of Microorganisms in the Selection of Reductive Activity Applied to a Ketone Mixture: Unexpected Highlighting of an Enantioselective Oxidative Activity
Source: Microorganisms. 2023 May 27;11(6):1415. doi: 10.3390/microorganisms11061415 (PMC10305621; doi:10.3390/microorganisms11061415)
Supplement: Supplementary file 1 [file microorganisms-11-01415-s001.zip › microorganisms-2362045-supplementary.pdf]

Combinatory library of microorganisms in the selection of reductive activity applied to a ketone mixture: highlighting of an enantioselective oxidative activity.

Sofiane Ali Rachedi, Maximillien Genest, Stéphane Mann and Didier Buisson

Unité Molécules de Communication et Adaptation des Microorganismes
Muséum National d'Histoire Naturelle, CNRS UMR 7245 CP54
57 rue Cuvier (63 rue Buffon) 75005 Paris, France

*** Correspondence:**
Didier Buisson
didier.buisson@mnhn.fr

Table S1. Wet weight of harvested biomass

Figure S1. GC chromatogram of mixture of authentic sample of substrates and their reduction products.

Figure S2. Chromatograms of phenylethanol on chiral column

Table S2. Analysis of biotransformation of ketones by CLMs

Table S1. Wet weight (in g) of harvested and incubated biomass

Strains	Weight of harvested biomass	Weight of incubated biomass
Candida magnolia MUCL27871	2.08	0.347
Cryptococcus humicola MUCL 30305	5.05	0.842
Kluyveromyces dobzanskii	2.01	0.670
Cryptococcus macerans MUCL 30616	0.87	0.145
Rhodotorula buffonii MUCL 29812	3.03	0.505
Pichia guilliermondii MUCL 27758	2.25	0.750
Rhodosporium toruloides MUCL 30328	2.97	0.495
Saccharomyces uvarum NRRL Y969	2.03	0.338
Sporidiobolus johnsonii ATCC 20490	1.89	0.630
Zygosaccharomyces fermentati MUCL 31281	2.22	0.370
Rhodotorula minuta MUCL 30637	3.94	0.657
Rhodotorula mucilaginosa	3.13	1.043
Pichia minuta MUCL 27758	3.25	0.542
Rhodosporidium toruloides MUCL 30010	1.44	0.240
Pichia anomala NRRL Y40	1.99	0.663
Kloekera magna NRRL Y1611	1.29	0.215
Torulaspora delbruckii MUCL 27816		
Candida pinus MUCL 27856	2.09	0.697
Saccharomyces montanus CBS 6772	1.80	0.300
Saccharomyces sp.	1.87	0.312
Rhodotorula rubra ATCC 4055	1.68	0.560
Rhodotorula pilimanae MUCL 27811	1.57	0.262
Rhodotorula glutinis NRRL 41091	1.55	0.258
Yarrowia lipolytica MUCL 29853		

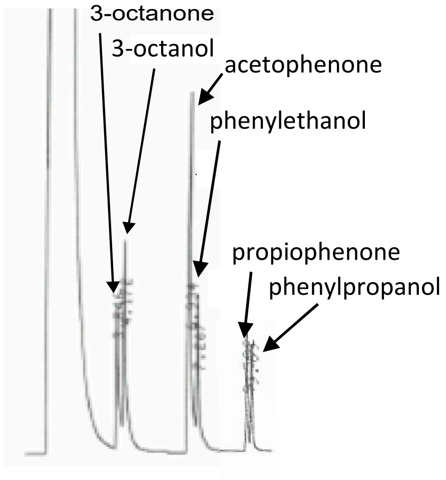

Fig. S1. GC chromatogram of mixture of authentic sample of substrates and their reduction products.

Conditions: Column OV1701 (Pierce, 15 m x 0.20 ram) capillary column, with helium (10 Psi) as a carrier gas, 90°C (5 min) then 90-120°C (5°C/min).

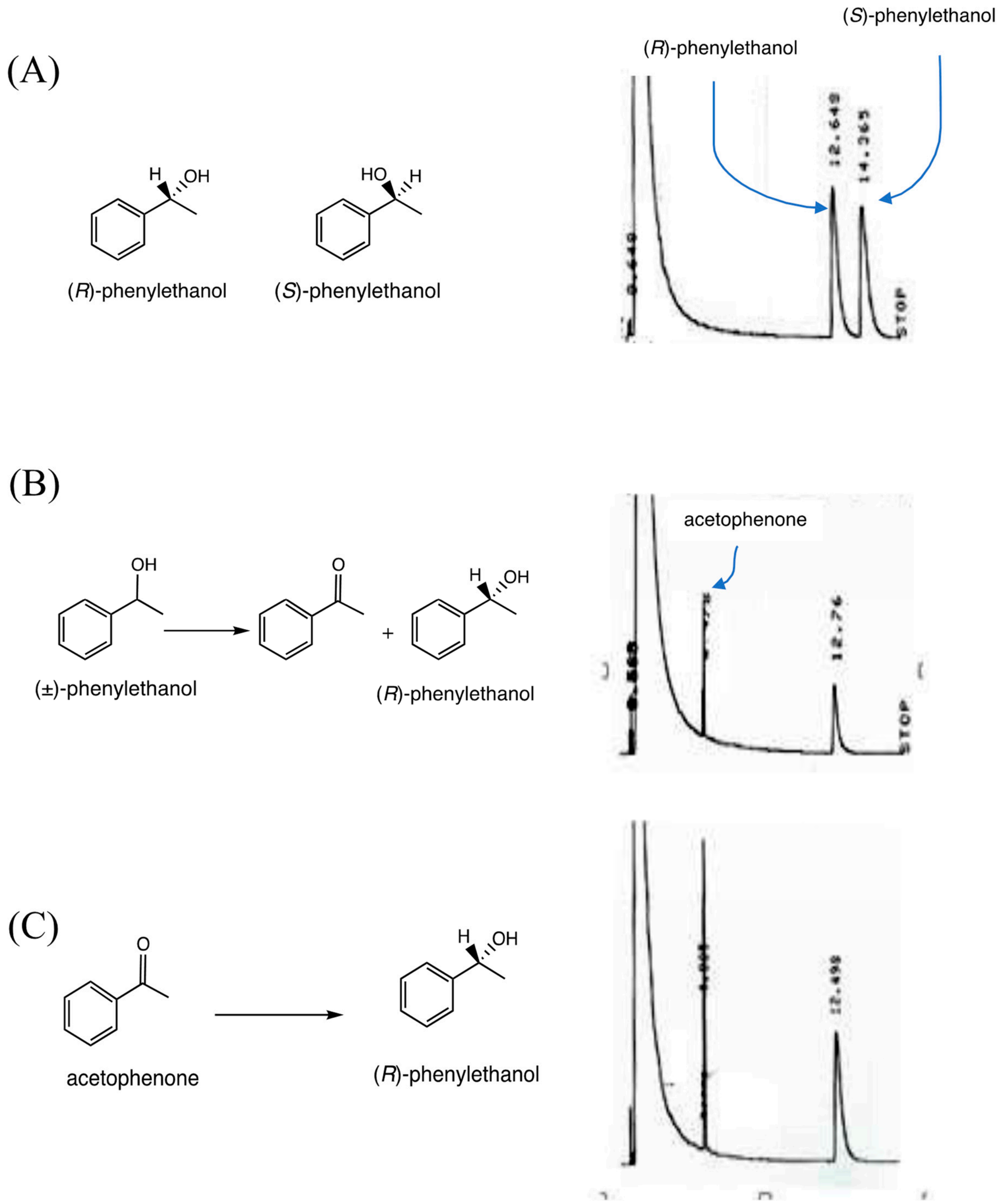

Fig. S2. Chromatograms of phenylethanol on chiral column

A: racemic mixture

B: Residual phenylethanol after 24h of incubation time with *R. buffoni* in aerobic conditions

C: Phenylethanol produced by reduction of acetophenone by *R. buffoni* in aerobic conditions after 24h of incubation time

Conditions: column CP-chirasil-Dex CB 25 m 0.32 mm, 25 µm, carrier gas He 15 Psi, oven temperature 100°C.

Table S2: Analysis of biotransformation of ketones by CLMs. Colour indicates the progress of the reaction (conversion %): blue: c <50%, purple: 50<c< 80 red: c > 80%

CLM	Time (h)	2-octanone 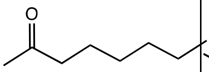	3-octanone 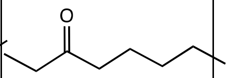	Acetonaphtone 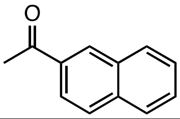	Propiophenone 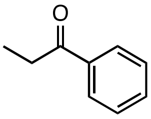	Acetophenone 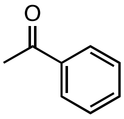
1	4	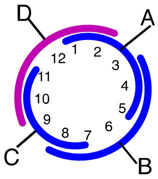	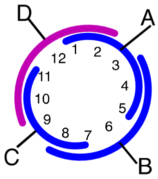	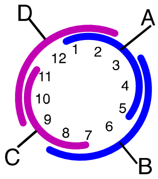	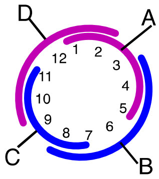	
	24	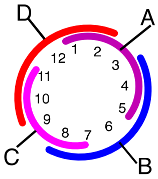	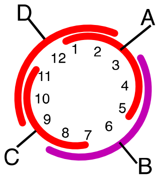	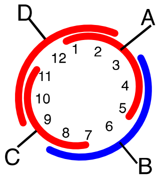	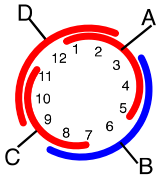	
2	4		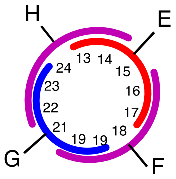			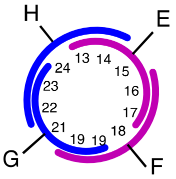
	24		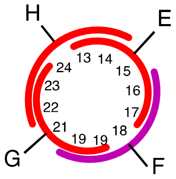			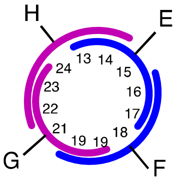
